# Unravelling the Role of Synthesis Conditions on the Structure of Zinc Oxide-Reduced Graphene Oxide Nanofillers

**DOI:** 10.3390/nano11082149

**Published:** 2021-08-23

**Authors:** Zélia Alves, Cláudia Nunes, Paula Ferreira

**Affiliations:** 1 Department of Chemistry, CICECO-Aveiro Institute of Materials, University of Aveiro, 3810-193 Aveiro, Portugal; zeliaralves@ua.pt; 2 Department of Materials and Ceramic Engineering, CICECO-Aveiro Institute of Materials, University of Aveiro, 3810-193 Aveiro, Portugal

**Keywords:** zinc oxide, reduced graphene oxide, solvothermal synthesis, structure, lattice defects

## Abstract

The diversity of zinc oxide (ZnO) particles and derived composites applications is highly dependent on their structure, size, morphology, defect amounts, and/or presence of dopant molecules. In this work, ZnO nanostructures are grown in situ on graphene oxide (GO) sheets by an easily implementable solvothermal method with simultaneous reduction of GO. The effect of two zinc precursors (zinc acetate (ZA) and zinc acetate dihydrate (ZAD)), NaOH concentration (0.5, 1 or 2 M), and concentration (1 and 12.5 mg/mL) and pH (pH = 1, 4, 8, and 12) of GO suspension were evaluated. While the ZnO particle morphology shows to be precursor dependent, the average particle size length decreases with lower NaOH concentration, as well as with the addition of a higher basicity and concentration of GO suspension. A lowered band gap and a higher specific surface area are obtained from the ZnO composites with higher amounts of GO suspension. Otherwise, the low concentration and the higher pH of GO suspension induce more lattice defects on the ZnO crystal structure. The role of the different condition parameters on the ZnO nanostructures and their interaction with graphene sheets was observed to tune the ZnO–rGO nanofiller properties for photocatalytic and antimicrobial activities.

## 1. Introduction

Nanofillers have become interesting structures to enhance the performance properties of eco-friendly biopolymer-based materials and further enhance its functionality [1,2]. Among the type of nanofillers, inorganic and carbon-based structures, particularly zinc oxide (ZnO) nanoparticles [3] and reduced graphene oxide (rGO) [4] have been explored.

ZnO is a low toxic metal oxide that has been receiving particular attention in the food packaging field not only to improve the mechanical and barrier properties [5], but also due to its unique features, namely UV light barrier [6], antioxidant activity [7], and especially its antimicrobial property even in the dark against several foodborne bacteria, including *Staphylococcus aureus* and *Escherichia coli* [8]. This set of properties, together with its abundance, biocompatibility, and reasonable stability at high temperatures and pressures, are additional positive points [9]. Moreover, ZnO particles are considered food grade to be used in food contact materials such as plastics, with a migration limit of 25 mg/kg of food [10]. Regarding the rGO, this carbon-based material has shown considerable importance due to its mechanical and thermal properties, but also because of its exceptional electrically properties and antioxidant activity [11,12].

The combination of ZnO and rGO as a composite is an appealing approach to prepare an active agent with unique features, since synergic mechanisms can potentiate their functional properties [13]. Nowadays, the ZnO–rGO composites have been explored in gas sensing, photocatalysis, and energy applications [14,15,16]. Different synthetic methodologies have been reported to prepare the ZnO–rGO materials, but the hydrothermal/solvothermal approach can be distinguished due to its highly efficiency, simplicity, low cost, and environmental friendly nature, since it does not require hazardous organic solvents [17,18]. This methodology consists in using water or ethanol as a solvent, where the nanoparticles are formed under a relatively low temperature and at high pressure inside a Teflon autoclave for a specific time [19]. By this green and one-pot approach, the synthesis and growth of ZnO particles can occur attached and/or wrapped to the rGO sheets, simultaneously with the reduction of graphene oxide [20]. As reported before, the hydrothermal treatment of GO favoured its reduction into rGO [21]. The synthesis of composite materials based on ZnO–rGO enables to obtain ZnO particles with different sizes and morphologic characteristics [18,22,23]. Although the detailed mechanisms remain controversial, the antibacterial activity of ZnO seems to exhibit a favourable performance when the particles have a high surface area and smaller particle size, with the nano ZnO being more promising than micro ZnO [24,25,26,27]. Therefore, the control of ZnO morphology has received great attention since shows different effects on the antibacterial activity [24,28,29]. For example, a study found that the flower-like ZnO nanoparticles exhibited a higher antibacterial activity than the rod- and sphere-like ZnO [28]. The authors claim this result due to a larger content of oxygen vacancy on the surface of the ZnO flower-like [28]. Thus, besides the size and morphology of ZnO particles, the enhancement of surface interstitial defects (oxygen vacancies and zinc interstitial) should not be neglected since these are reported to have a considerable effect on the antimicrobial properties. These defects reduce the electrons or holes recombination and promote the generation of reactive oxygen species (ROS), which are a possible mechanism for bacteria inactivation [30,31,32]. The defects structure of ZnO particles is also responsible for the antioxidant activity [33].

As the synthesis process has a strong influence on the final ZnO–rGO composite, a diversity of parameters should be controlled including, for instance, the type and concentration of zinc precursor [34], pH of solution [35], temperature [36], time [37], and the variation of graphene oxide (GO) concentration [38]. The GO synthesis occurs mainly following the Hummers’ method where acidic solvents are employed [39], and even with an extensive washing process, it is very difficult to achieve a GO suspension with a neutral pH. To the best of our knowledge, the effect of pH of the GO suspension on the growth of ZnO particles has never been study as well as a combined study of GO suspension and pH variability of the reaction medium. The combination of ZnO with GO, under solvothermal conditions, causes some changes in the structure of each individual material mainly due to the association of ZnO with rGO sheets, where some functional groups of rGO (e.g., hydroxyl and carboxyl) can be incorporated in the interstitial sites of ZnO structures, affecting some optical and structural properties [17]. The changes in the size, morphology, and lattice of ZnO and on the reduction degree of rGO can affect the final functional properties of the ZnO–rGO composite. Therefore, the understanding of ZnO–rGO structure is of crucial importance to use it as a nanofiller.

This work is not only devoted to the synthesis and characterization of ZnO and ZnO–rGO nanocomposites but also aims to contribute to a deeper understanding of the rGO interaction with ZnO nanoparticles. To fulfil these aims a systematic variation of the solvothermal synthesis conditions was performed and correlated with the final structural, morphological, and optical properties of the nanofiller.

## 2. Materials and Methods

### 2.1. Reagents

Zinc acetate was purchased by Sigma-Aldrich and zinc acetate dihydrate by Panreac. Other reagents were analytical grade or higher available purity. Ethanol absolute anhydrous and sodium hydroxide were obtained from Carlo Erba reagents (Barcelona, Spain) and LabChem Inc (Zelienople, PA, USA), respectively. Graphite (~150 µm flakes), phosphoric acid (≥85%), hydrochloric acid (37%), potassium permanganate (99.0%), sulfuric acid (97%), hydrogen peroxide (30%), were purchased from Sigma-Aldrich Co. (St Lois, MO, USA).

### 2.2. Synthesis of Graphene Oxide

Graphene oxide (GO) was prepared by oxidation and exfoliation from pure graphite powder according to the improved modified Hummers’ method [39]. Firstly, 180 mL of H_2_SO_4_ and 20 mL of H_3_PO_4_ were added to graphite flakes (1.5 g) in a flask and mixed at room temperature. The KMnO_4_ (9 g) was added slowly under stirring, producing a slight exotherm reaction. The reaction was warmed to 50 °C in an oil bath and stirred for 16 h. The mixture was cooled to room temperature and 200 mL of ice were added. Then, 30% H_2_O_2_ was added to solubilize manganese ions from residual permanganate (the reaction colour turns yellow). The mixture was centrifuged (6000 rpm for 30 min), and the supernatant was separated and discarded. The remaining solid material was then washed successively with water, 30% HCl, and ethanol (twice) to remove the impurity ions. Finally, the material was dispersed in water to obtain a suspension and was exfoliated through ultrasonication for 45 min by probe sonication (45 W, pulse 10 s and pause 5 s), obtaining the GO suspension.

### 2.3. Solvothermal Synthesis of ZnO Particles and ZnO–rGO Composites

The nanoparticles were synthesized based on a solvothermal methodology previously reported [40]. Briefly, two solutions of 0.1 M of zinc precursor were prepared in ethanol, one of zinc acetate (ZA) (Zn(CH_3_COO)_2_) and another of zinc acetate dihydrate (ZAD) (Zn(CH_3_COO)_2_·2H_2_O). Sodium hydroxide (NaOH) solutions in ethanol were prepared with different concentrations (0.5, 1.0, and 2.0 M) (Table 1). The GO suspension was diluted in distilled water to obtain a final suspension with 1.0 mg/mL and 12.5 mg/mL. Then, the pH of GO suspension at 1.0 mg/mL were adjusted to 4 and 8, while at 12.5 mg/mL were adjusted to 1, 8, and 12. For 10 mL of ZA or ZAD solution were dripped 1.6 mL of GO suspension under stirring for 30 min. Afterwards, 20 mL of NaOH solution were added dropwise under vigorous stirring for 1 h. Synthesis of pure ZnO was also performed using the same procedure replacing the GO suspension by 1.6 mL of distilled water. The solutions were transferred to a Teflon-lined stainless-steel autoclave (60 mL) and heated in an oven at 150 °C for 24 h to produce ZnO nanostructures incorporated on reduced graphene oxide (rGO). The produced precipitates were collected, washed with water and ethanol several times until neutral pH, and then, were dried at 60 °C to be further characterized. Table 1 summarizes all the experiments performed. Samples of ZnO without GO addition are denoted as NaOH *^x y^*, where *x* indicates the zinc precursor, ZA or ZAD for zinc acetate or zinc acetate dihydrate, respectively, and *y* indicates the molarity of the NaOH solution (0.5, 1, or 2). Adding the GO suspension, the ZnO–rGO samples are named as NaOH *^x^* ^*y*^–GO *^z^* ^*w*^, where *x* and *y* have the same connotation described earlier, *z* indicates the GO concentration (1 or 12.5 for the 1 and 12.5 mg/mL), and *w* is referent to the pH of GO suspension (1, 4, 8, and 12).

### 2.4. Nanoparticles’ Characterization

Powder X-ray diffraction (XRD) was performed on a Panalytical Empyrean X-ray diffractometer (Malvern Panalytical, Malvern, UK) with Cu-Kα radiation (λ = 1.54178 Å). The diffractograms were recorded in a reflection mode with the scanning angle ranged from 5 to 70° 2*θ*. For phase identification, an integrated database of powder diffraction files (JCPDS) from the International Centre of Data Diffraction (ICDD) was employed.

Scanning electron microscopy (SEM) was employed to observe the morphology of as-prepared ZnO and ZnO–rGO nanostructures using a field-emission gun SEM Hitachi SU70 microscope (Hitachi High-Tech Corporation, Tokyo, Japan) operated at 15 kV. Samples were deposited on a sample holder and coated with evaporated carbon.

The BET (Brunauer–Emmett–Teller) method was used for the calculation of the specific surface area. The −196 °C nitrogen adsorption/desorption isotherms were acquired using a Micromeritics^®^–Gemini 2380 V2.00 (Micromeritics Instrument Corporation, Norcross, GA, USA) and the samples degassed at 120 °C overnight. Raman spectroscopy was carried out using Jobin Yvon T64000 instrument (HORIBA, Kyoto, Japan) equipped with a laser operating at 441 nm as an excitation source wavelength laser.

The Fourier transform infrared (FTIR) spectra were acquired using a Golden Gate single reflection diamond attenuated total reflectance (ATR) system in a Bruker IFS-55 (Billerica, MA, USA) spectrometer. Spectra were recorded at the absorbance mode at a wavenumber range from 4000 to 400 cm^−^^1^ (mid-infrared region) with a resolution of 4 cm^−^^1^. Five replicates (64 co-added scans) were collected for each sample.

Ultraviolet/visible (UV/Vis) diffuse reflectance spectroscopy (DRS) was recorded using a UV/vis spectrophotometer Cintra 101 (GBC Scientific equipment Ltd., Dandenong, Victoria, Australia), with a wavelength range from 200–800 nm and a resolution of 0.5 nm. BaSO_4_ powder was used as reference for baseline measurements.

## 3. Results and Discussion

The synthesis of ZnO structures was previously described to be very susceptible to different conditions, from the type of zinc precursor to the pH of solution and to the presence of different molecules in the medium such as the GO [34,35,38]. Throughout this work the effects of these parameters on the ZnO structures are explored, but also on the reduction of GO. For a clearer comprehensive look, the results and discussion are sectioned in three parts: (i) effect of zinc precursor, NaOH concentration and GO addition on ZnO structures; (ii) effect of GO concentration on ZnO structures; and (iii) effect of pH of GO suspension on ZnO structures. The surface morphology and structure of ZnO and ZnO–rGO particles were mainly characterized by SEM, FTIR, XRD, and RAMAN, and some of them by UV-vis DRS.

### 3.1. Effect of Zinc Precursor, NaOH Concentration and GO Addition on ZnO Structures

The synthesis of ZnO and ZnO–rGO composites was studied with two different zinc precursors, ZA and ZAD, three concentrations of NaOH (0.5, 1.0, and 2.0 M), which originate solutions with different final pH, and evaluating the presence of GO suspension (1.0 mg/mL). Figure S1 presents the XRD patterns of the ZnO and ZnO–rGO structures synthetized. The major diffraction peaks of nanostructures were observed at 2*θ* values around 31.8°, 34.5°, 36.3°, 47.6°, 56.6°, 62.9°, 66.4°, 67.9°, 69.1°, corresponding to (100), (002), (101), (102), (110), (103), (200), (112) and (201) crystalline planes, respectively. These crystalline planes are consistent to the hexagonal phase of wurtzite structure of ZnO particles and corresponds to P*63*mc space group as seen by comparison to the representative peaks from powder diffraction files database (JCPDS) card no 36–1451. The presence of sharp, intense, and well-defined peaks is a good evidence of the crystallinity of the synthetized ZnO nanostructures. Moreover, no other crystalline planes were observed indicating the synthesis of monophasic ZnO crystalline structures.

The addition of GO suspension does not led to the appearance of the characteristic diffraction peaks of GO in the ZnO–rGO diffractograms for both precursors, which could be an indication of the GO reduction under solvothermal conditions. Moreover, the high crystallinity of ZnO nanoparticles, the restricted restacking of rGO sheets in presence of ZnO, the extensive exfoliation of rGO, and the low GO concentration can justify the absence of the typical rGO reflections in the XRD patterns.

**Figure 1:**
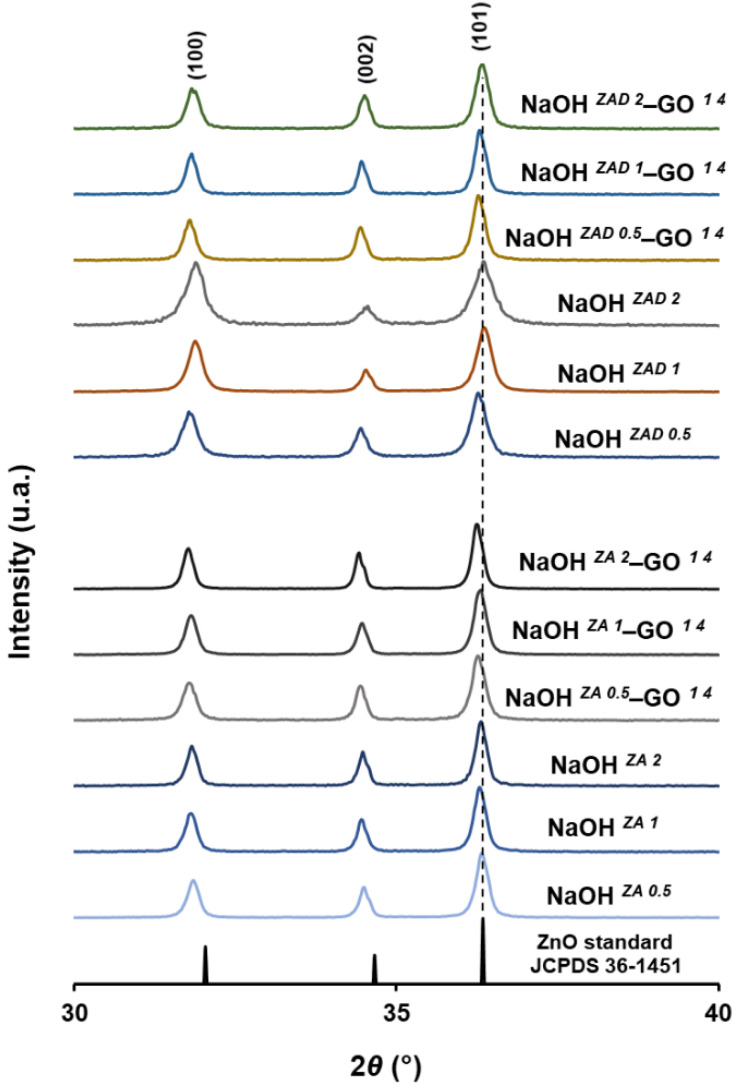
XRD diffractograms of ZnO and ZnO–rGO composites varying the NaOH concentration (0.5, 1, and 2 M) and the zinc precursor (ZA and ZAD).

For all the ZnO and ZnO–rGO nanostructures, the most prominent reflection at the XRD patterns corresponds to the (101) plane, indicating the preferred orientation. Concerning this diffraction plane, more information about the prepared nanostructures can be obtained, particularly the full width half maximum (FWHM) and the 2*θ* diffraction angle (Table 2). For all the nanostructures synthetized from ZA precursor, the FWHM value drops slightly when the NaOH concentration increases. The same trend occurs in the FWHM value of synthetized structures using the ZAD precursor, but only up to 1.0 M NaOH concentration, the most alkaline syntheses demonstrated an increase in the FWHM values. In general, the data demonstrate that increasing the pH of synthesis solution with higher concentrations of NaOH results in lower FWHM value, which is indicative that the crystal quality of ZnO and ZnO–rGO nanostructures is significantly improved. Other authors also reported a decrease of FWHM with increasing the pH of the reaction medium from 4 to 8, claiming a crystallite size growth of ZnO nanoparticles at the higher pH values [41]. When using the ZAD precursor, it is observed from XRD analysis (Figure 1) that the diffraction peak position (2*θ*) shifts towards higher angles upon increasing the NaOH concentration, with or without the presence of graphene sheets. However, the same behaviour is not so obvious with the nanostructures obtained from the ZA precursor. The shifting of peaks towards higher angles revealed a decrease in lattice parameters (Table 2) of ZnO when increasing the NaOH concentration solution added to the reaction medium. A reported study about pure ZnO also showed the shift of XRD peaks to higher 2*θ* values when the pH of precursors solution is increased [42]. Comparing the samples obtained with the same precursor and NaOH concentration, the addition of GO suspension shift the peaks to lower angles, except for the NaOH *^ZA 1^*–GO *^1 4^* and NaOH *^ZAD 0.5^*–GO *^1 4^* samples. This is an indication that ZnO crystal lattice is changed through the association with the graphene sheets. In fact, peak position shifted towards lower 2*θ* values was also observed through the doping of ZnO with N ions or carbon quantum dots, where the ions can substitute the O sites or being inserted in the interstitial sites in ZnO crystal lattice [43,44]. Thus, a kind of interaction, like Zn-O-C, can occur between the ZnO and the functional groups of graphene sheets, since the shift is also followed with the increase of ZnO lattice parameters (Table 2). Both NaOH concentration and GO suspension seem to have a relevant influence on changing the ZnO crystal lattice sites.

**Figure 2:**
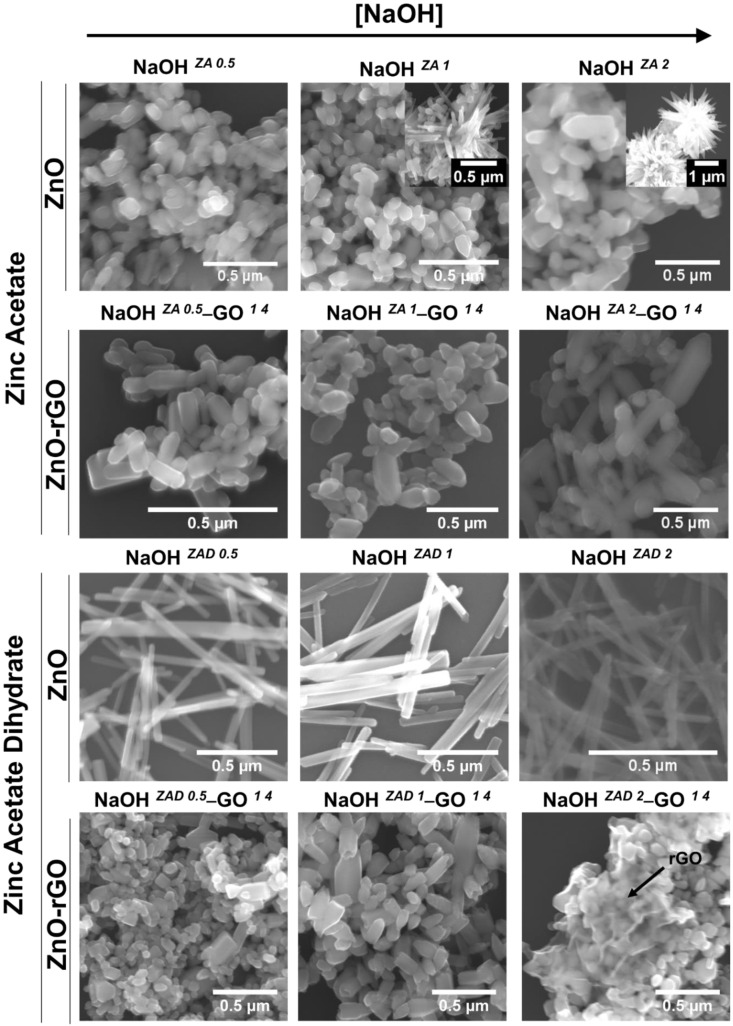
SEM micrographs of ZnO and ZnO–rGO composites obtained by solvothermal synthesis using zinc acetate anhydride and dehydrated and NaOH concentrations of 0.5, 1.0 and 2.0 M. The inset images at NaOH *^ZA 1^* and NaOH *^ZA 2^* samples represent additional morphologies of ZnO particles with anisotropic growth directions.

The morphology and size of ZnO and ZnO–rGO structures were characterized by scanning electron microscopy (SEM) and the micrographs are displayed in Figure 2.

Regarding the ZA precursor, a tendency of ZnO structures aggregation occurred for all the NaOH concentrations studied. There is a mixture of morphologies of hexagonal or spherical shape, but the most prevalent is the nanorod-like structure. However, increasing the concentration of NaOH to 1.0 and 2.0 M results in heterogeneous size and morphology of ZnO particles, with the nanorods converted to flower-like structures, showing more anisotropic growth directions. The synthesis of ZnO using ZAD as a precursor showed a thin ZnO nanorod structure in all the concentrations of NaOH. Comparing the anhydrous and hydrate zinc precursors, different morphologies were obtained for the same solution conditions, having the ZnO structures produced with ZAD a higher aspect ratio. These changes in the morphology could be related with precursor solubility in ethanol [45], since zinc acetate has lower solubility (0.5 g/100 mL) than zinc acetate dihydrate (3 g/100 mL). The coordination of zinc acetate with water molecules increases its solubility which enable the faster growth of ZnO particles along to the *c*-axis.

The particle size length (Figure S2) of ZnO nanostructures using the ZA precursor increase with increasing NaOH concentration, being more accentuated at 2.0 M NaOH. For ZAD precursor, the growth occurs up to 1.0 M NaOH, above this concentration the particle size length decreases. These results showed that particle size is dependent of pH and of the precursor employed in the synthesis solution.

In the presence of GO suspension, the morphology of ZnO particles is similar for both zinc precursors independently of NaOH concentration. From SEM micrographs (Figure 2), the ZnO structures are nanorods with different aspect ratio, indicating that there are structures in different growth phases. The particle size length of ZnO in the ZnO–rGO structures provided from the ZA precursor is not much changed when compared with the pristine ZnO nanoparticles, and also demonstrated a gradually growth with the increase of NaOH concentration (Figure S2). What stands out with the addition of GO suspension is the growth inhibition of ZnO particles using ZAD as zinc precursor. In fact, the size of ZnO particles using ZAD is greatly reduced, but their growth shows the same behaviour observed in the absence of GO, i.e., the maximum growth size is achieved with 1.0 M NaOH. The reduction of GO during the solvothermal methodology allows to produce ZnO structures with a low aspect ratio when compared with the synthesis without the addition of GO. This observation is in agreement with Feng et al. [46] who also reported that the addition of graphene sheets changes the ZnO morphology from micrometre rods to nanoparticles. In fact, the oxygenated groups present on the GO surface increase the nucleation sites to anchor the Zn^2+^ ions by chemical coordination, as already observed in other studies with ZnO [47] but also in other metal oxides such as TiO_2_ [48]. Consequently, the availability of Zn^2+^ ions to the ZnO particle growth is decreased, which enables to a reduction of their size [49]. GO sheets are visible in the synthesis with ZAD and 2.0 M NaOH (Figure 2), but the same did not occur using ZA as zinc precursor. This could occur because of the low concentration of GO, but also because of ZnO structures being completely anchored on the surface of GO sheets, making it impossible to be seen.

### 3.2. Effect of GO Concentration on ZnO Structures

The further characterization is related with the ZnO and ZnO–rGO structures synthesized from ZA precursor with NaOH concentration at 2.0 M, varying the concentration of GO suspension (1.0 mg/mL and 12.5 mg/mL). The pH of GO suspension was previously corrected to 8.

**Figure 3:**
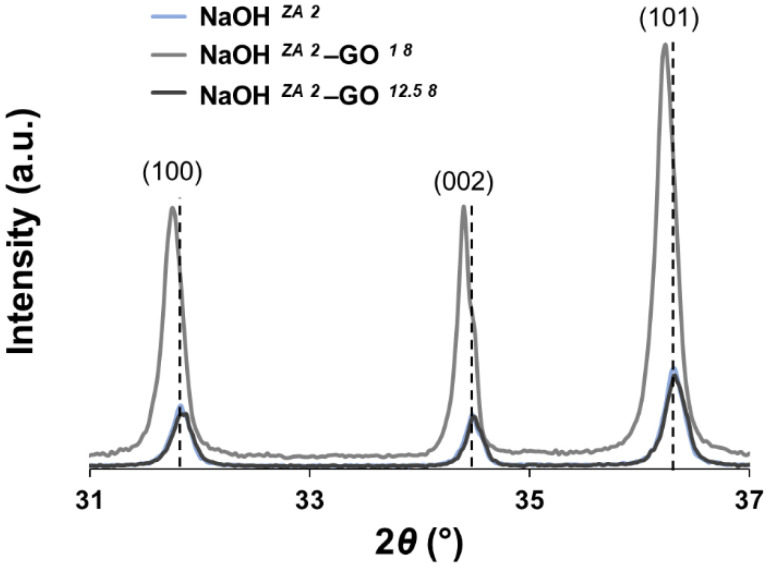
XRD diffractograms of ZnO (NaOH *^ZA 2^*) and ZnO–rGO structures (NaOH *^ZA 2^*–GO *^1 8^* and NaOH *^ZA 2^*–GO *^12.5 8^*) in the 2*θ* range from 31 to 37°.

XRD was employed to characterize the influence of the two different concentrations of GO suspension in the crystal structure of ZnO particles (Figure 3). The three most intense peaks of the XRD patterns, corresponding to the (100), (002), and (101) planes, are consistent with the hexagonal wurtzite phase of pristine ZnO. Furthermore, the absence of additional planes in the diffraction data confirms that the crystallinity and preferred orientation of ZnO is not changed with the GO addition. A shift to lower angles as well as a slight increase in the lattice parameters (Table 3) are observed in the NaOH *^ZA 2^*–GO *^1 8^* sample when compared to the NaOH *^ZA 2^*, but the increase of GO concentration in the composite does not produce the same effect. These results can indicate that in the presence of low amounts of GO occurs a coordination between the ZnO and the oxygen groups of graphene sheets, enabling different strains in the ZnO lattice. Evaluating the XRD in more detail, the FWHM value of the highest intense peak (101) increases with the higher GO content, which suggests that rGO affects the growth of ZnO crystallites.

**Figure 4:**
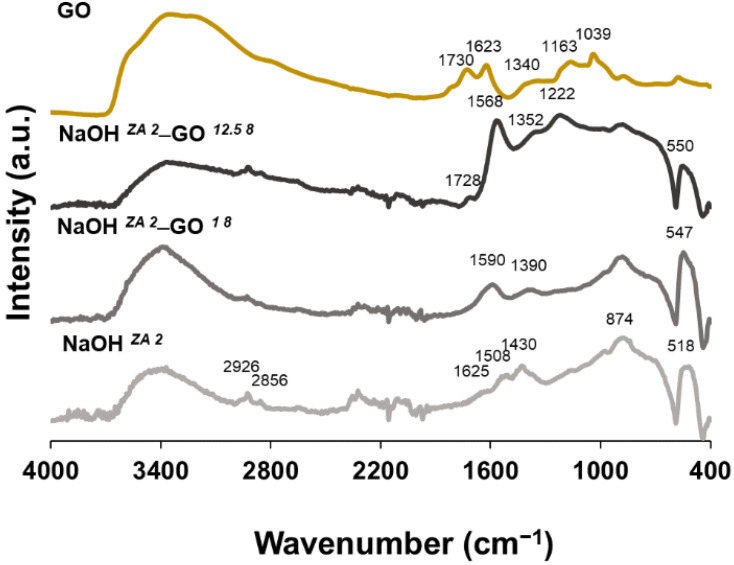
ATR-FTIR spectra of GO, ZnO (NaOH *^ZA 2^*) and ZnO–rGO structures varying the concentration of GO suspension: 1 mg/mL (NaOH *^ZA 2^*–GO *^1 8^*) and 12 mg/mL ((NaOH *^ZA 2^*–GO *^12.5 8^*).

**Figure 5:**
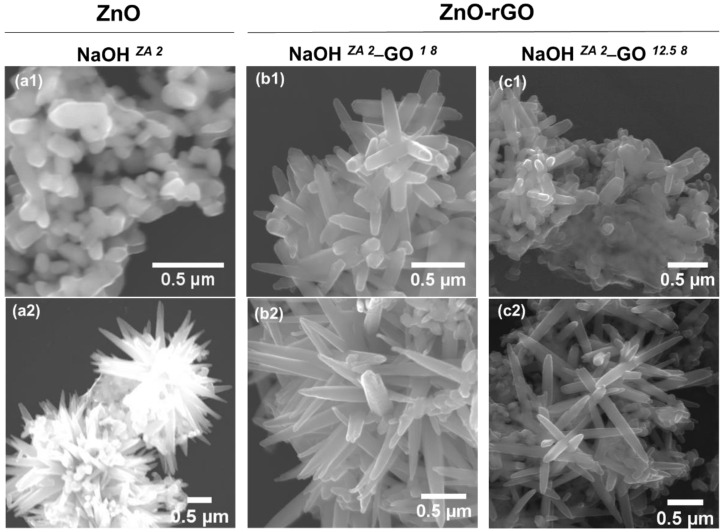
SEM micrographs of ZnO (NaOH *^ZA 2^* (**a1**,**a2**)) and ZnO–rGO structures varying the concentration of GO suspension: 1 mg/mL (NaOH *^ZA 2^*–GO *^1 8^* (**b1**,**b2**)) and 12.5 mg/mL (NaOH *^ZA 2^*–GO *^12.5 8^* (**c1**,**c2**)).

Figure 4 shows the characteristic functional groups recorded by ATR-FTIR for the ZnO, ZnO–rGO nanostructures, and GO samples. Looking to the NaOH *^ZA 2^* sample, a broad peak at 3630–3000 cm^−^^1^ and a weak band at 1625 cm^−^^1^ are observed, corresponding to –OH stretching vibration of water molecules absorbed onto the surface [50]. The observed peaks at 2926 and 2856 cm^−^^1^ are assigned to the sp^3^ and sp^2^ carbon stretching vibrations, respectively. Additionally, the bands at 1508 and 1430 cm^−^^1^ can be attributed to the carbonyl groups of residual acetate groups of zinc precursor [51,52]. Peak at 879 cm^−^^1^ and the high intense one at 518 cm^−^^1^ occur due to the metal-oxygen vibrational modes of ZnO compounds. However, the last peak is shifted in the composites to 547 and 550 cm^−^^1^ for the NaOH *^ZA 2^*–GO *^1 8^* and NaOH *^ZA 2^*–GO *^12.5 8^*, respectively, verifying a perturbation in the Zn–O–Zn network with the addition of GO and the increase of its concentration. These findings corroborate the XRD data, which also deduced a coordination of ZnO with the functional groups of graphene sheets, as observed in other studies [53,54]. In the case of GO, a broad band with a maximum at 3350 cm^−1^ results from the presence of –OH groups as well as from adsorbed water molecules. A GO surface rich in –OH groups can be useful to anchor other materials on its surface, such as ZnO particles. In addition, GO also shows the following characteristic bands at 1730 cm^−^^1^ (carboxylate C=O stretching), 1623 cm^−^^1^ (C=C bond), 1340 cm^−^^1^ (carboxyl C–O, epoxide C–O–C or phenolic C–OH), and 1039 cm^−^^1^ (alkoxy C–O) [55,56]. The peak intensities associated with the oxygen functional groups are decreased or disappear in the spectrum of ZnO–rGO nanostructures, indicating the reduction of GO sheets and the interaction with the ZnO particles. From NaOH *^ZA 2^*–GO *^12.5 8^* spectrum, it is observed that bands related with the stretching vibrations of C=O (1728 cm^−^^1^), C–OH (1362 cm^−^^1^), and epoxy C–O–C (1222 cm^−^^1^) decrease in intensity and are shifted comparing to GO. In addition, weak aromatic C=C bonds of skeletal ring vibrations from the graphitic domain (1568 cm^−^^1^) have high intensity which should result from the restoration of sp^2^ linkages. All these bands are related to the presence of rGO [55,57,58,59]. Contrarily, NaOH *^ZA 2^*–GO *^1 8^* spectrum does not show the characteristic C=O and epoxy C–O–C bands, and the peaks related to C=C and C–OH are shifted to higher wavenumbers, which could indicate that GO has a higher percentage of reduction but also a higher anchoring of ZnO structures on graphene sheets [53]. The peaks related to the oxygen-functional groups are present in higher intensity in the NaOH *^ZA 2^*–GO *^12.5 8^* sample due to the higher weight percentage of GO added to the composite that was not fully reduced. This FTIR interpretation indicates that the interaction of ZnO structures with the graphene sheets is dependent of the GO concentration added to zinc precursor solution. Looking at the SEM micrographs (Figure 5), it is observed that the addition of GO suspension at 1.0 mg/mL, with the pH adjusted to 8, increases the length of ZnO nanorods (Figure 5(b1)) but, increasing the GO concentration to 12.5 mg/mL, the length of ZnO nanorods attached to the graphene is reduced (Figure 5(c1)) (Figure S3). Additionally, anisotropic growth directions are observed for the ZnO particles in all of the samples (Figure 5(a2,b2,c2)). These observations demonstrate that GO concentration has a great influence in the length size of ZnO particles, tuning the dimensional ZnO nanostructures from smaller to larger. The highest GO concentration results in smaller ZnO particles dispersed on the surface of rGO sheets due to the higher availability of nucleation sites.

**Figure 6:**
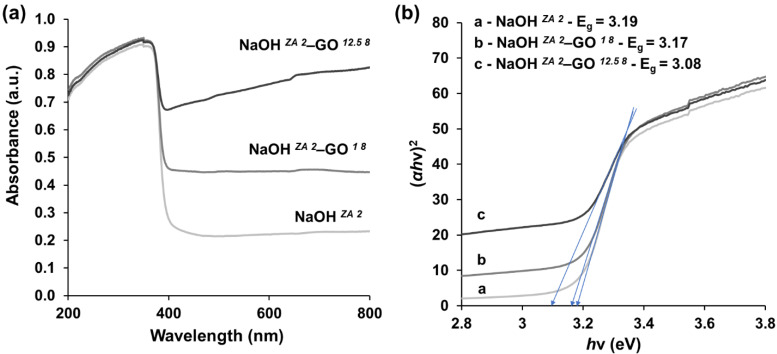
(**a**) UV-vis diffuse reflectance (DRS) and (**b**) Tauc plots of ZnO (NaOH *^ZA 2^*) and ZnO–rGO composites with the addition of different GO concentration: 1 mg/mL (NaOH *^ZA 2^*–GO *^1 8^*) and 12.5 mg/mL (NaOH *^ZA 2^*–GO *^12.5 8^*).

The optical properties of ZnO (NaOH *^ZA 2^*) and ZnO–rGO composites varying the loading amount of GO (NaOH *^ZA 2^*–GO *^1 8^* and NaOH *^ZA 2^*–GO *^12.5 8^* ) were characterized by UV-vis diffusion reflectance spectroscopy (DRS) (Figure 6). The absorption in the UV region is observed in all of the samples, as a result of the presence of ZnO particles, while the absorbance in the visible light is significantly higher with the increase of rGO content in the composite (Figure 6a), in agreement with the previous reports [17,60]. The high absorption in the visible light can occur due to the presence of rGO sheets since this material has a high absorption coefficient in the visible region. When rGO is present, the surface electric charge associated to the oxides in the ZnO–rGO composites is increased and alter the process of electron-hole pair formation during light irradiation [17,61]. The combination of strong absorption in UV and visible light is an important characteristic for the photocatalytic activity. From the spectra, it is observed that the absorption edge is slightly red shifted in the ZnO–rGO composites compared to pure ZnO particles, which implies a corresponding decrease in the band gap energy of the ZnO–rGO composite. The direct energy band gap of the samples was calculated according to the Tauc’s equation (Figure 6b). The band gap of pure ZnO was estimated to be ∼3.19 eV, a value close to the reported in the literature [62], and for ZnO–rGO was 3.17 eV and 3.08 eV with the addition of 1.0 and 12.5 mg/mL of GO suspension, respectively. The observed reduction in the band gap energy occurs with the incorporation of rGO and the reduction is more prominent in higher concentrations of rGO in the composite, mainly due to the formation of Zn-O-C chemical bonds in the ZnO–rGO composites [55,57]. In addition, the UV-vis DRS spectra confirm the synthesis of ZnO–rGO composites since the results show both the absorption of ZnO and rGO phases.

The values of surface area were estimated by Brunauer–Emmett–Teller (BET) and determined by low temperature N_2_ adsorption–desorption isotherms (Table 3). The pure ZnO (NaOH *^ZA 2^*) and the ZnO–rGO composites (NaOH *^ZA 2^*–GO *^1 8^* and NaOH *^ZA 2^*–GO *^12.5 8^*) have a surface area of 7.09, 10.76, and 14.61 m^2^/g, respectively. The data show that the specific surface area increases in the ZnO–rGO composites and reaches higher values upon increasing the GO content. The improvement of the BET surface area in the composites can be attributed to the rGO incorporation, which is characterized with a large surface area. In addition, the active surface sites of graphene sheets prevent the agglomeration of the large-size ZnO particles, and hence increasing the BET surface area [63]. The improvement of this parameter is beneficial for the photocatalytic performance [63,64] and antimicrobial activity [65].

**Figure 7:**
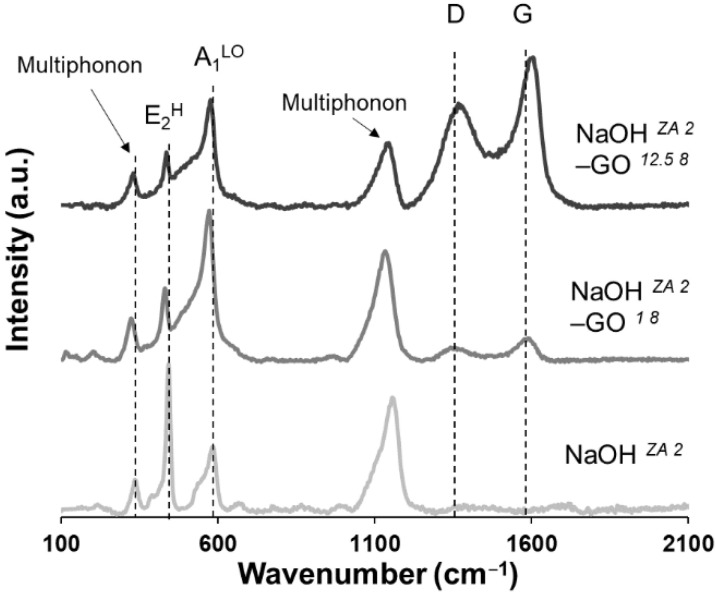
Raman spectra of ZnO (NaOH *^ZA 2^*) and ZnO–rGO composites with the addition of different GO concentration: 1 mg/mL (NaOH *^ZA 2^*–GO *^1 8^*) and 12.5 mg/mL (NaOH *^ZA 2^*–GO *^12.5 8^*).

Raman spectroscopy was done to comprehend the role of GO content on the vibrational properties of ZnO, identifying some structural disorder and defects that are present in the ZnO structures (Figure 7). The modes around 336, 441, 583, and 1155 cm^−^^1^ are visible in all the three samples and are attributed to E_2_^H^-E_2_^L^ (multiphonon scattering), E_2_^H^, A_1_^LO^, and 2A_1_^LO^ and 2E_1_^LO^ (multiphonon), respectively [66]. These ZnO peak positions are significantly shifted to lower wavenumbers with the addition of GO, with the maxima position shifts corresponding to the NaOH *^ZA 2^*–GO *^1 8^* sample. The signature E_2_^H^ mode, attributed to the oxygen atoms [67], appears in the NaOH *^ZA 2^* sample as the peak with the highest intensity and with the lowest FWHM value, while the increase of GO concentration gradually decreases the peak intensity and widening of its FWHM (Table S1). Moreover, the E_2_^H^ peak confirms the wurtzite structure in all samples in consistence with the XRD results. Although the presence of this peak is an intrinsic characteristic of wurtzite hexagonal phase of ZnO, and represents its higher crystallinity, the results suggest a weak ZnO crystalline structure with the increase of GO concentration, due to a possible substitution of the GO oxygen-functional groups in the ZnO lattice. A previously study also reported the decrease of peak intensity and an increase of FWHM with the enhancement of Co doping concentrations in ZnO [68]. Additionally, the A_1_^LO^ peak, attributed to the impurities and structural defects, such as dislocation and oxygen-zinc vacancy states [38,67], is shifted in the presence of GO. This result suggests that ZnO–rGO composites have more lattice defects than pure ZnO, being more intense in the NaOH *^ZA 2^*–GO *^1 8^* sample. In fact, this sample demonstrates the highest intensity of A_1_^LO^ mode and its highest shifting, which suggests that the lower addition of GO concentration induces more lattice defects in the crystal structure. These results are consistent with the XRD data, since the defects of ZnO particles in the composite with lower addition of GO amount are attributed to surface impurities or the presence of dopants such the graphene sheets attached to the ZnO particles [69]. It is worth noting that the intensity ratio of E_2_^H^ over A_1_^LO^ is greater in the NaOH *^ZA 2^* sample than in the composites, where the intensity ratio decreases due to the inverse peak intensity. Therefore, the combined effect of the suppressed intensity of E_2_^H^ peak with the higher intensity of A_1_^LO^ mode indicates that the presence of GO enhances the defect states of ZnO particles. In addition to the peaks related with ZnO structures, two other important peaks appear in the composites corresponding to the D (≈1363 cm^−^^1^) and G (≈1598 cm^−^^1^) band, both characteristic of carbon components in graphitic materials. While the first one is attributed to the defects or disorders in the hexagonal graphitic structure, the second one is related to the sp^2^ hybridized carbon [70]. The simultaneous presence of ZnO and graphitic peaks confirms the effective preparation of ZnO–rGO composites. The intensity of both D and G bands is higher in the NaOH *^ZA 2^*–GO *^12.5 8^* sample due to the higher GO amount in the composite. The I_D_/I_G_ ratio is lower in the NaOH *^ZA 2^*–GO *^1 8^* sample (0.89) than on the NaOH *^ZA 2^*–GO *^12.5 8^* (1.36) (Table S1), which indicates an increase of structural defects in the rGO lattice with the enhancement of GO amount along with the higher ratio of Zn^2+^ ions interactions with GO sheets [17].

### 3.3. Effect of pH of GO Suspension on ZnO Structures

**Figure 8:**
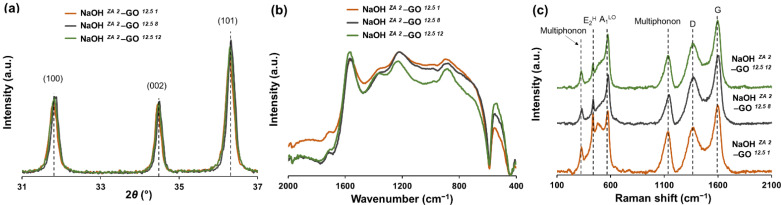
XRD (**a**), FTIR (**b**), and Raman (**c**) results of ZnO–rGO composites varying the pH of GO suspension to 1 (NaOH *^ZA 2^*–GO *^12.5 1^*), 8 (NaOH *^ZA 2^*–GO *^12.5 8^*), and 12 (NaOH *^ZA 2^*–GO *^12.5 12^*).

**Figure 9:**
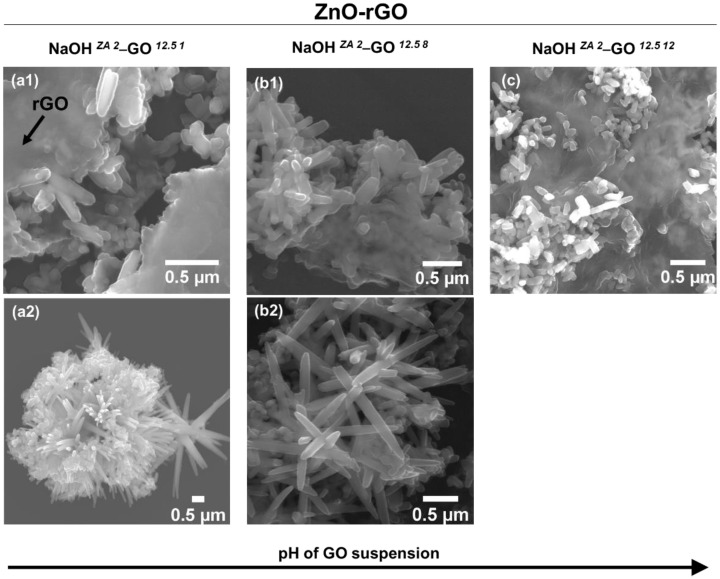
SEM images of ZnO–rGO composites varying the pH of GO suspension to 1 (NaOH *^ZA 2^*–GO *^12.5 1^* (**a1**,**a2**)), 8 (NaOH *^ZA 2^*–GO *^12.5 8^* (**b1**,**b2**)), and 12 (NaOH *^ZA 2^*–GO *^12.5 12^* (**c**)).

In this section, samples synthesized using the ZA precursor with 2M NaOH and adding GO suspension (12.5 mg/mL) at pH 1, 8, and 12 were characterized in terms of XRD, FTIR, RAMAN and SEM. From XRD spectra (Figure 8a), all samples have the same diffraction pattern representative of the ZnO hexagonal wurtzite structure, suggesting that the initial pH difference in GO suspension is negligible in the structure of ZnO particles. Despite the lack of significant changes in XRD patterns, ATR-FTIR absorption (Figure 8b) of NaOH *^ZA 2^*–GO *^12.5 12^* sample shows a shift to higher wavenumbers in the peak related to the C–OH (1364 cm^–^^1^) and C–O–C (1231 cm^−^^1^) when compared with the samples with GO suspension at pH 1 and 8 (C–OH (1352 cm^−^^1^) and C–O–C (1223 cm^−^^1^)). These shifts in peak positions can be explained by structural variations that take place in the vicinity of each functional group presented in the graphene sheets, more specifically structural changes in the basal plane, but these can also occur due to interactions with ZnO particles. Moreover, the intensity of peak related with the Zn–O–Zn vibrations increases with the pH enhancement of GO suspension, revealing that the anchoring of ZnO particles to the rGO sheets is influenced with the initial pH of GO suspension. Increasing the GO suspension basicity, the carboxyl and hydroxyl groups resulted from the oxidation process are deprotonated, acting as nucleation sites for the growth of ZnO particles. As the degree of deprotonation is different among the samples, the strains of ZnO particles are also different. This can be confirmed by the Raman spectra (Figure 8c) where the characteristic peaks related with the ZnO particles undergo changes due to the pH variation of GO suspension. When GO suspension turns alkaline, the FWHM value related to the E_2_^H^ peak is enhanced and the ratio of E_2_^H^ and A_1_^LO^ intensities is decreased (Table 4). This means that increasing the pH of GO suspension, the defects states of ZnO are more pronounced, resulting also in the loss of higher crystallinity. Concerning the rGO structure (Table 4), the ratio of I_D_/I_G_ slightly increases with the alkalinity of GO suspension, indicating that rGO has more defects disorder. Besides that, NaOH *^ZA 2^*–GO *^12.5 12^* sample also shows a FWHM of D band larger than in the other samples, that associated to the FTIR results, corroborated more defects or disorders in the basal structure or the association between the oxygen functional groups with the ZnO particles. Looking at the SEM images of ZnO–rGO composites (Figure 9), the ZnO particles are morphologically such as rod-shaped, but in both conditions with lower pH it is possible to see ZnO particle clusters that are not associated to the graphene sheets (Figure 9(a2,b2)). In addition, the average of ZnO size length and its dispersion decreased with the basicity of GO suspension (Figure S4). As described before, the deprotonation of functional groups of GO with the pH increment enhances the nucleation sites of Zn^2+^ in the graphene sheets which decreases the zinc precursor availability to increase the growth of ZnO particles. In contrast, when the functional groups of GO are protonated, the particle growth is promoted.

## 4. Conclusions

This work allowed a comprehensive overview on the synthesis of ZnO and ZnO–rGO structures where a variety of conditions is reviewed with the use of a solvothermal methodology, using ethanol as a solvent. The results confirm the successful production in all the synthesis of ZnO particles with the wurtzite hexagonal structure. Under the same synthesis conditions, the addition of GO (1 mg/mL) to the ZA and ZAD precursor produced ZnO structures with a similar nanorod-like morphology, while the absence of GO showed nanorod and flower-like structures for the ZnO using the ZA precursor and thin nanorod with high aspect ratio using ZAD precursor. The average length of ZnO can also be controlled. The ZnO particle size is lower with a low NaOH concentration, and with the increase of concentration and basicity of GO suspension. The functionality of ZnO–rGO composites, due to the higher specific surface area and lower band gap, can be improved if the synthesis is performed with the GO suspension at 12.5 mg/mL and pH adjusted to 12. The defects amount on the ZnO structure can also be optimized by controlling the GO suspension conditions. At pH 8, the increase of defect states is potentiated with the low amount of GO because there is a higher ratio of Zn^2+^ over GO sheets that encourages the interaction of the ZnO particles with the rGO sheets. In addition, the highest defect amounts on the ZnO structures are achieved increasing the basicity of GO suspension. In this condition, the oxygen-containing groups resulted from the oxidation process are deprotonated and act as nucleation sites for the growth of ZnO nanofillers, promoting their anchoring in a higher extent over the graphene surface. The findings of this study provide a fundamental insight into how the ZnO structures can be tailored/modulated by adjusting the different conditions of synthesis, i.e., the type of zinc precursor, NaOH concentration, and concentration and pH of GO suspension. This knowledge is fundamental to control the required ZnO and ZnO–rGO properties in according to their potential application as a nanofiller for photocatalytic and/or antimicrobial activities.

## Figures and Tables

**Table 1:** Conditions of synthesis of ZnO and ZnO–rGO nanostructures: (i) zinc acetate precursors with different degrees of hydration (ZA and ZAD); (ii) presence or absence of GO at 1 mg/mL or 12.5 mg/mL; (iii) varying the NaOH concentration; and (iv) changing the pH of GO suspension.

Precursor	NaOH (M)	(GO) (mg/mL)	pH of GO	Sample ID
ZA (Zn(CH_3_COO)_2_)	0.5	1.0	4	NaOH *^ZA 0.5^*–GO *^1^*^*4*^
		-	-	NaOH *^ZA 0.5^*
	1.0	1.0	4	NaOH *^ZA 1^*–GO *^1^*^*4*^
		-	-	NaOH *^ZA 1^*
	2.0	1.0	4	NaOH *^ZA 2^*–GO *^1^*^*4*^
			8	NaOH *^ZA 2^*–GO *^1^*^*8*^
		12.5	1	NaOH *^ZA 2^*–GO *^12.5^*^*1*^
			8	NaOH *^ZA 2^*–GO *^12.5^*^*8*^
			12	NaOH *^ZA 2^*–GO *^12.5^*^*12*^
		-	-	NaOH *^ZA 2^*
ZAD (Zn(CH_3_COO)_2_·2H_2_O)	0.5	1.0	4	NaOH *^ZAD 0.5^*–GO *^1^*^*4*^
		-	-	NaOH *^ZAD 0.5^*
	1.0	1.0	4	NaOH *^ZAD 1^*–GO *^1^*^*4*^
		-	-	NaOH *^ZAD 1^*
	2.0	1.0	4	NaOH *^ZAD 2^*–GO *^1^*^*4*^
		-	-	NaOH *^ZAD 2^*

**Table 2:** Peak position of (101) plane present in the XRD diffractograms of ZnO and ZnO–rGO composites varying the NaOH concentration and the zinc precursor. The respective lattice parameters and the FWHM are also presented.

	2*θ* (°)	Lattice Parameters (Å)		FWHM
		a = b	c	
NaOH *^ZA 0.5^*	36.3157	3.2403	5.1927	0.2442
NaOH *^ZA 1^*	36.2894	3.2456	5.2003	0.2408
NaOH *^ZA 2^*	36.3157	3.2429	5.1965	0.2258
NaOH *^ZA 0.5^*–GO *^1^*^*4*^	36.2632	3.2482	5.2003	0.2657
NaOH *^ZA 1^*–GO *^1^*^*4*^	36.3157	3.2429	5.1965	0.2422
NaOH *^ZA 2^*–GO *^1^*^*4*^	36.242	3.2482	5.2042	0.2139
NaOH *^ZAD 0.5^*	36.2632	3.2482	5.2003	0.3108
NaOH *^ZAD 1^*	36.3733	3.2377	5.1888	0.3022
NaOH *^ZAD 2^*	36.3719	3.2377	5.1850	0.4304
NaOH *^ZAD 0.5^*–GO *^1^*^*4*^	36.2632	3.2456	5.2003	0.2503
NaOH *^ZAD 1^*–GO *^1^*^*4*^	36.2894	3.2429	5.2003	0.2320
NaOH *^ZAD 2^*–GO *^1^*^*4*^	36.3419	3.2429	5.1926	0.2685

**Table 3:** The FWHM value and lattice parameters of (101) plane present in the XRD diffractograms of ZnO and ZnO–rGO structures. The BET value of the samples is also represented.

	FWHM	Lattice Parameters (Å)		BET (m^2^/g)
		a = b	c	
NaOH *^ZA 2^*	0.22583	3.243	5.193	7
NaOH *^ZA 2^*–GO *^1^^8^*	0.23691	3.251	5.208	11
NaOH *^ZA 2^*–GO *^12.5^^8^*	0.25299	3.243	5.193	15

**Table 4:** Raman data related with the modes of ZnO and rGO of ZnO–rGO composites varying the pH of GO suspension to 1 (NaOH *^ZA 2^*–GO *^12.5 1^*), 8 (NaOH *^ZA 2^*–GO *^12.5 8^*), and 12 (NaOH *^ZA 2^*–GO *^12.5 12^*).

	E_2_^H^ FWHM	E_2_^H^/A_1_^LO^	FWHM		I_D_/I_G_
			D	G	
NaOH *^ZA 2^*–GO *^12.5^^1^*	17.758	0.956	165	90	1.32
NaOH *^ZA 2^*–GO *^12.5^^8^*	23.027	0.525	165	88	1.36
NaOH *^ZA 2^*–GO *^12.5^^12^*	32.696	0.482	170	88	1.38

## Data Availability

The data is included in the main text and/or the supplementary materials.

## Supplementary Materials

The following are available online at https://www.mdpi.com/article/10.3390/nano11082149/s1, Figure S1: XRD diffractograms of ZnO and ZnO–rGO composites varying the NaOH concentration (0.5, 1, and 2 M) and the zinc precursor (ZA and ZAD), Figure S2: Boxplot representing the variation of ZnO particle size length as a function of NaOH concentration (0.5 M, 1.0 M, and 2.0 M), for the two zinc acetate precursors (ZA and ZAD) in the presence or absence of graphene oxide, Figure S3: Particle size length of ZnO (NaOH *^ZA 2^*) and ZnO-rGO structures varying the concentration of GO suspension: 1 mg/mL (NaOH *^ZA 2^*–GO *^1 8^*) and 12.5 mg/mL (NaOH *^ZA 2^*–GO *^12.5 8^*), Table S1: Raman data of ZnO (NaOH *^ZA 2^*) and ZnO–rGO structures (NaOH *^ZA 2^*–GO *^1 8^* and NaOH *^ZA 2^*–GO *^12.5 8^*) related with the modes of ZnO and rGO, Figure S4: Particle size length of ZnO structures on the ZnO–rGO composites varying the pH of GO suspension to 1 (NaOH *^ZA 2^*–GO *^12.5 1^*), 8 (NaOH *^ZA 2^*–GO *^12.5 8^*), and 12 (NaOH *^ZA 2^*–GO *^12.5 12^*).
