# Supplementary material for: Unravelling the Role of Synthesis Conditions on the Structure of Zinc Oxide-Reduced Graphene Oxide Nanofillers
Source: Nanomaterials (Basel). 2021 Aug 23;11(8):2149. doi: 10.3390/nano11082149 (PMC8399407; doi:10.3390/nano11082149)
Supplement: Supplementary file 1 [file nanomaterials-11-02149-s001.zip › nanomaterials-1350979-supplementary.pdf]

nanomaterials

Supplementary Materials

Unravelling the Role of Synthesis Conditions on the Structure of Zinc Oxide-Reduced Graphene Oxide Nanofillers

Zélia Alves [1,2], Cláudia Nunes [1,*] and Paula Ferreira [2,*]

[1] Department of Chemistry, CICECO-Aveiro Institute of Materials, University of Aveiro, 3810-193 Aveiro, Portugal; zeliaralves@ua.pt
[2] Department of Materials and Ceramic Engineering, CICECO-Aveiro Institute of Materials, University of Aveiro, 3810-193 Aveiro, Portugal
* Correspondence: claudianunes@ua.pt (C.N.); pcferreira@ua.pt (P.F.)

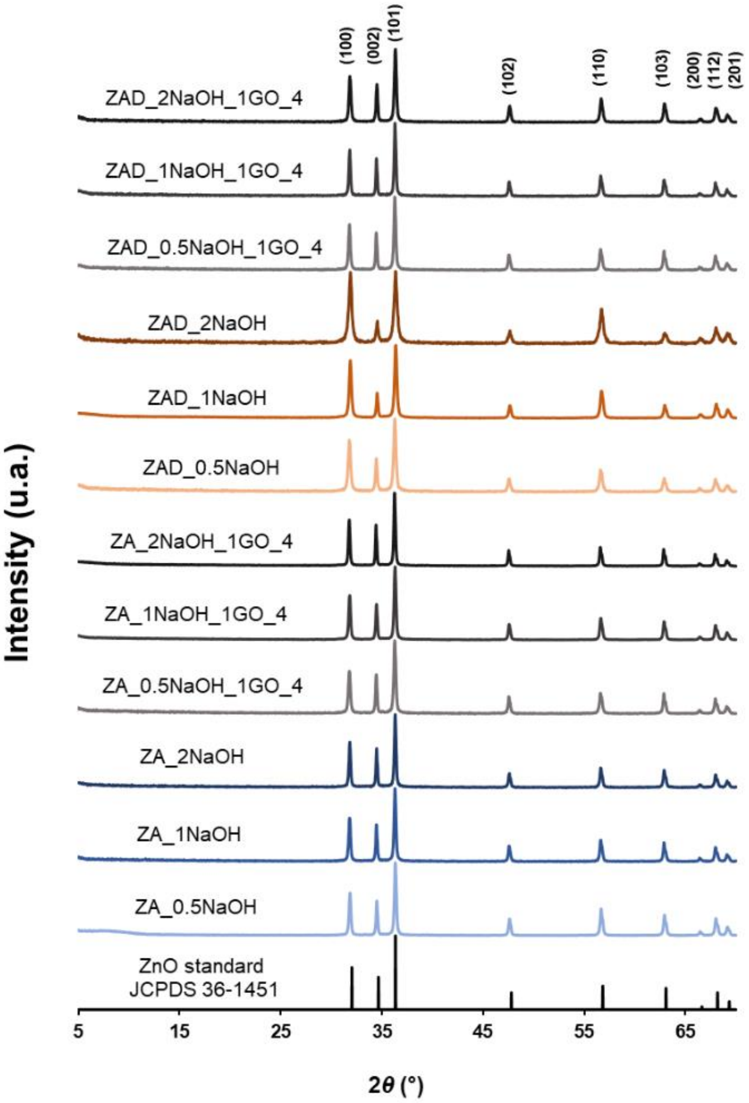

Figure S1. XRD diffractograms of a) ZnO and ZnO-rGO composites varying the NaOH concentration (0.5, 1, and 2 M) and the zinc precursor (ZA and ZAD).

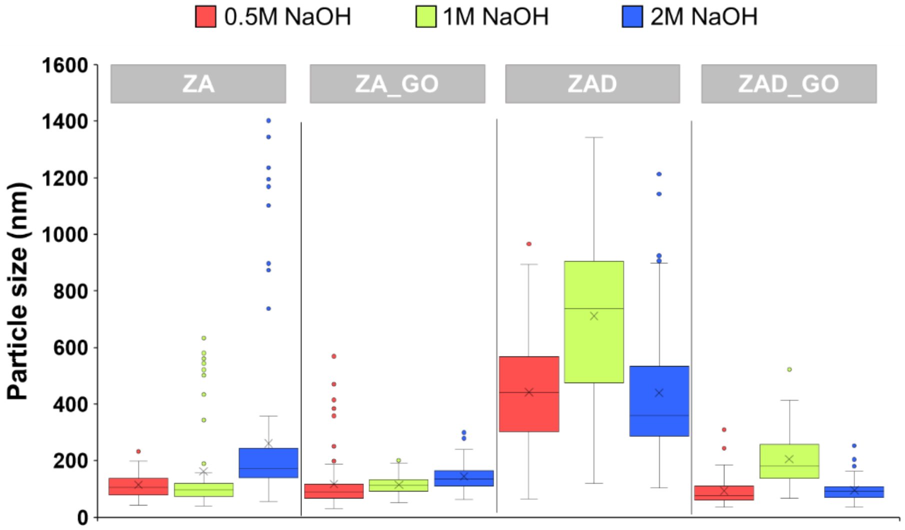

Figure S2. Boxplot representing the variation of ZnO particles size as a function of NaOH concentration (0.5 M, 1.0 M, and 2.0 M), for the two zinc acetate precursors in the presence or not of graphene oxide. (ZA – Zinc Acetate, ZA_GO – Zinc Acetate with the addition of graphene oxide, ZAD – Zinc Acetate Dihydrate, ZAD_GO – Zinc acetate dihydrate with the addition of graphene oxide).

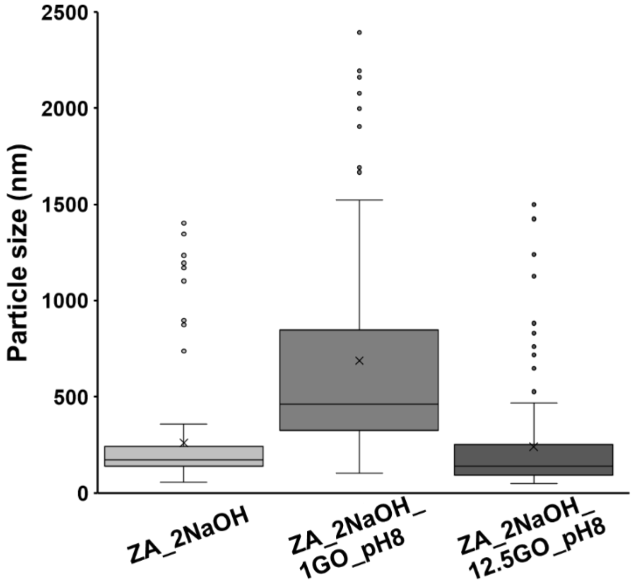

Figure S3. Particle size of ZnO and ZnO-rGO structures varying the concentration of GO solution: ZA_2NaOH, ZA_2NaOH_1GO_8, and ZA_2NaOH_12.5GO_8.

Table S1. Raman data of ZA_2MNaOH, ZA_2MNaOH_1GO_pH8, and ZA_2MNaOH_12.5GO_pH8 samples related with the modes of ZnO and rGO.

	E_2^H FWHM	E_2^H/A_1^{LO}	FWHM		I_D/I_G
			D	G	
ZA_2NaOH	15.322	2.174	-	-	-
ZA_2NaOH_1GO_8	22.975	0.493	167	69	0.89
ZA_2NaOH_12.5GO_8	23.027	0.525	165	88	1.36
